# Serotonergic Antidepressants and Risk for Traumatic Intracranial Bleeding

**DOI:** 10.3389/fneur.2021.758707

**Published:** 2021-10-28

**Authors:** Harri Isokuortti, Grant L. Iverson, Jussi P. Posti, Jori O. Ruuskanen, Antti Brander, Anneli Kataja, Milaja Nikula, Juha Öhman, Teemu M. Luoto

**Affiliations:** ^1^Department of Neurosurgery, Helsinki University Hospital and University of Helsinki, Helsinki, Finland; ^2^Department of Physical Medicine and Rehabilitation, Harvard Medical School, Center for Health and Rehabilitation Research, Spaulding Rehabilitation Hospital and Spaulding Research Institute, Home Base, A Red Sox Foundation and Massachusetts General Hospital Program, Charlestown, MA, United States; ^3^Department of Neurosurgery, Neurocenter, Turku Brain Injury Center, Turku University Hospital and University of Turku, Turku, Finland; ^4^Division of Clinical Neurosciences, Department of Neurology, Turku University Hospital and University of Turku, Turku, Finland; ^5^Medbase Ltd., Turku, Finland; ^6^Department of Radiology, Medical Imaging Centre, Tampere University Hospital, Tampere, Finland; ^7^Faculty of Medicine and Life Sciences, University of Tampere, Tampere, Finland; ^8^Department of Neurosurgery, Tampere University Hospital, Tampere, Finland; ^9^Department of Neurosurgery, Tampere University Hospital and Tampere University, Tampere, Finland

**Keywords:** brain injuries, traumatic, intracranial hemorrhages, antidepressant agents, anticoagulation, antithrombotic agents (MeSH)

## Abstract

**Background:** Serotonergic antidepressants may predispose to bleeding but the effect on traumatic intracranial bleeding is unknown.

**Methods:** The rate of intracranial bleeding in patients with antidepressant medication was compared to patients not antidepressants in a cohort of patients with acute head injury. This association was examined by using a consecutive cohort of head trauma patients from a Finnish tertiary center emergency department (Tampere University Hospital, Tampere, Finland). All consecutive (2010–2012) adult patients (*n* = 2,890; median age = 58; male = 56%, CT-positive = 22%, antithrombotic medication users = 25%, antidepressant users = 10%) who underwent head CT due to head trauma in the emergency department were included.

**Results:** Male gender, GCS <15, older age, and anticoagulation were associated with an increased risk for traumatic intracranial bleeding. There were 17.8% of patients not taking antidepressants and 18.3% of patients on an antidepressant who had traumatic intracranial bleeding (*p* = 0.830). Among patients who were taking antithrombotic medication, 16.6% of the patients not taking antidepressant medication, and 22.5% of the patients taking antidepressant medication, had bleeding (*p* = 0.239). In a regression analysis, traumatic intracranial hemorrhage was not associated with antidepressant use.

**Conclusions:** Serotonergic antidepressant use was not associated with an increased risk of traumatic intracranial hemorrhage.

## Introduction

Intracranial bleeding is the most severe and feared complication of head trauma. Hemorrhagic lesions may be life-threatening, require urgent neurosurgical care, and they might cause long-term disability. Several risk factors for intracranial bleeding following head trauma include age, high-energy trauma mechanism, fall from a height, history of coagulopathy, and use of anticoagulants (1–5).

Antidepressants, especially selective serotonin reuptake inhibitors (SSRIs), are a commonly used group of medications. The prevalence of antidepressant use has been estimated to be between 2.7 and 15.7% in the adult population in the European Union and United States (6, 7). There is a concern that SSRIs have been linked to increased risk of bleeding, including hemorrhagic stroke (8–18). This increase in bleeding risk is thought to be due to the role of serotonin in platelet aggregation, which is inhibited by SSRIs, as well as to a direct decrease in platelet adhesion to both collagen and fibrinogen (9, 19–21)—important processes in the initiation of hemostasis.

The possible association between SSRI use and risk for intracranial bleeding is not well-understood. Theoretically, small subcortical microbleeds following shearing forces (acceleration/deceleration) to the brain might enlarge in the setting of compromised platelet function associated with use of SSRIs. The objective of this study was to investigate the risk for intracranial hemorrhage in patients on serotonergic antidepressant therapy who present to the emergency department with head trauma and undergo computed tomography (CT). We hypothesized that the use of serotonergic antidepressants would be associated with an increased risk for intracranial hemorrhages following head injury.

## Materials and Methods

### Study Setting and Ethics

The current study included patients initially enrolled in the Tampere Traumatic Head and Brain Injury Study (study code: NCT01427959). All patients were from the ED of the Tampere University Hospital (Tampere, Finland). The ED provides health services for a joint municipal authority of 22 municipalities (both urban and rural) with a total of approximately 470,000 residents. The Tampere University Hospital is the main trauma center and only neurosurgical referral hospital in the hospital district. In this study, the minimum criteria for traumatic brain injury (TBI) were defined as follows: either blunt injury to the head or acceleration/deceleration type injury resulting in an initial Glasgow coma scale (GCS) score of 13–15, witnessed loss of consciousness, disorientation, or amnesia. These criteria were used to include patients with a TBI and to exclude patients with an isolated head injury without signs of TBI. The endpoint of the study was acute intracranial traumatic hemorrhage visible on the primary CT scan.

This study was approved by the Ethics Committee of Pirkanmaa Hospital District, Tampere, Finland (identifier: R10027). Institutional ethics and research board approval was also obtained.

### Study Sample

The participants were enrolled during a 2-year period between August 2010 and July 2012. The patients in this cohort consisted of all consecutive patients who underwent head CT due to acute head trauma (*n* = 3,023). A detailed retrospective data collection was conducted on demographics, injury-related data, premorbid health, medication, clinical characteristics, and neuroimaging findings. In this retrospective sample, referral criteria for acute head CT were based on the Scandinavian Guidelines for Initial Management of Minimal, Mild, and Moderate Head Injuries from 2000 (22). The data collection has been described in detail in previous publications (23, 24). For the present study, patients under 16 years (*n* = 133) were excluded and in total 2,890 adult patients were included.

### Medication Data

Antidepressants that were considered serotonergic and in use in Finland were: (i) citalopram, (ii) escitalopram, (iii) sertraline, (iv) fluoxetine, (v) paroxetine, (vi) venlafaxine, (vii) duloxetine, (vii) vortioxetine, (viii) amitriptyline, (ix) nortriptyline, (x) doxepin, and (xi) trimipramine. Drugs were further grouped into SSRIs, serotonin-norepinephrine reuptake inhibitors (SNRIs), and tricyclic antidepressants (TCAs) based on their structure and pharmacodynamic properties.

Information on the current use of serotonergic antidepressants was collected from the hospital records. Antithrombotic medication use was also recorded. Antiplatelet medications included acetylsalicylic acid (ASA), ASA-dipyridamole, dipyridamole, ticlodipine, clopidogrel, prasugrel, and ticagrelor. Anticoagulant medication included warfarin, apixaban, dabigatran, edoxaban, and rivaroxaban, and low-molecular weight heparins (LMWH).

### Imaging

All of the head CT scans were interpreted by a board certified neuroradiologist and systematically coded using an independent coding protocol (24). The cohort was collected before the Common Data Elements (CDEs) for TBI imaging by the National Institute of Neurological Disorders and Stroke were established (25). However, all CDEs possible with non-contrast structural CT scan were included in the neuroradiological case report form. The focus of this study was on hemorrhagic head CT lesions (epidural hematoma, subdural hematoma, subarachnoid hemorrhage, contusion, intracerebral hemorrhage, and intraventricular hemorrhage).

### Statistical Analysis

Dichotomous variables were compared using the chi-square test for proportions and continuous variables using the Mann-Whitney U-test. Interactions between antithrombotic use and antidepressant use were analyzed with two-way ANOVA. Unconditional logistic regression modeling was performed to estimate the odds ratios (ORs) and 95% CIs of acute intracranial hemorrhage in patients on serotonergic antidepressants while controlling for multiple possible confounders (GCS, antiplatelet medication, anticoagulation, gender, and age). The regression model confounders were selected based on their clinical relevance in relation to demographics, TBI severity, and intracranial hemorrhage risk. The level of statistical significance was set at 5%. SPSS Statistics for Windows (IBM Corp., Armonk, NY, USA) was used for data analyses. The STROBE guidelines were followed (26).

## Results

The characteristics of the study patients are presented in Table 1. The median age of the patients was 58.4 years. The most common injury mechanism was a ground-level fall. Antithrombotic medication was used by 24.6%. The most common anticoagulant was warfarin, and the most used antiplatelet medication was ASA.

**Table 1:** Characteristics of the study sample.

**Variable**	**All CT-scanned patients** ***n*** **=** **2,890**		**CT-scanned patients on serotonergic antidepressants** ***n*** **=** **287**		**CT-scanned patients without serotonergic antidepressants** ***n*** **=** **2,603**		***p*-Value**
Age (years)	Md = 58.4	Range = 16.0–103.8	Md = 68.7	Range = 16.4–94.6	Md = 57.4	Range = 16.0–103.8	<0.001
	***n***	**%**	***n***	**%**	***n***	**%**	
Men	1,619	56.0	179	62.3	1,092	42.0	<0.001
Women	1,271	44.0	108	37.6	1,511	58.0	
Diseases of the circulatory system	1,542	53.4	122	42.5	1,420	54.6	<0.001
Mental and behavioral disorders	774	26.8	154	53.7	620	23.8	<0.001
Diseases of the nervous system	711	24.6	120	41.8	591	22.7	<0.001
Injury mechanism							
Unknown	72	2.5	6	2.1	66	2.5	0.646
Car accident	286	9.9	11	3.8	275	10.6	<0.001
Ground-level fall	1,563	54.1	218	76.0	1,345	51.7	<0.001
Motorcycle accident	49	1.7	2	0.7	47	1.8	0.167
Bicycle accident	116	4.0	7	2.4	109	4.2	0.152
Fall from a height	301	10.4	21	7.3	280	10.8	0.070
Sports	54	1.9	1	0.3	53	2.0	0.045
Violence	218	7.5	12	4.2	206	7.9	0.023
Other, not specified	175	6.1	9	3.1	166	6.4	0.029
Any antithrombotic medication	710	24.6	121	42.2	589	22.6	<0.001
Anticoagulant medication	330	11.4	40	13.9	290	11.1	0.157
Warfarin	329	11.4	40	13.9	289	11.1	0.151
Apixaban	0	0	0	0.0	0	0.0	N/A
Dabigatran	1	0.0	0	0.0	1	0.0	0.740
Rivaroxaban	0	0	0	0.0	0	0.0	N/A
Low molecular weight heparin	21	0.7	2	0.7	19	0.7	0.951
Antiplatelet medication	398	13.8	85	29.6	313	12.0	<0.001
Acetylsalicylic acid	329	11.4	74	25.8	255	9.8	<0.001
• Acetylsalicylic acid-dipyridamol	47	1.6	8	2.8	39	1.5	0.101
Clopidogrel	24	0.8	4	1.4	20	0.8	0.268
Ticagrelor	0	0	0	0.0	0	0.0	N/A
• Acetylsalicylic acid and clopidogrel	9	0.3	1	0.3	8	0.3	0.906
Dipyridamole	8	0.3	0	0.0	8	0.3	0.347
Glasgow coma scale score 15	1,509	52.2	146	51.2	1,363	52.4	0.102
Glasgow coma scale score 13–14	130	4.4	7	2.4	123	4.7	0.142
Glasgow coma scale score 3–12	244	8.4	19	6.6	225	8.6	0.434
Loss of consciousness, witnessed	549	19.0	30	10.5	519	19.9	<0.001
Post-traumatic amnesia	646	22.4	55	19.2	591	22.7	0.172

*CT, computed tomography; GCS was categorized as above in order to form subgroups of similar size*.

The traumatic lesions are reported in Table 2. The most common hemorrhagic lesion was subdural hematoma. There were 17.8% of patients not taking antidepressants and 18.3% of patients on an antidepressant who had traumatic intracranial bleeding (*p* = 0.830). Among patients who were taking antithrombotic medication, 16.6% of the patients not taking antidepressant medication, and 22.5% of the patients taking antidepressant medication, had bleeding (*p* = 0.239).

**Table 2:** Types of traumatic head CT lesions.

	**Total sample** ***n*** **=** **2,890**		**On antidepressant** ***n*** **=** **287**		**No antidepressant** ***n*** **=** **2,603**		***p*-Value**
**Type of traumatic lesion**	***n***	**%**	***n***	**%**	***n***	**%**	
Skull fracture	157	5.4	11	3.8	146	5.6	0.208
Epidural hematoma	19	0.7	1	0.3	18	0.7	0.495
Subdural hematoma							
Subdural hematoma, acute	350	12.1	35	12.2	350	13.4	0.963
Subdural hematoma, chronic	100	3.5	9	3.1	91	3.5	0.751
Subdural hematoma, mixed density	11	0.4	1	0.3	10	0.4	0.926
Traumatic subarachnoidal hemorrhage	278	9.6	21	7.3	257	9.9	0.163
Contusion	216	7.5	25	8.7	191	7.3	0.401
Intraventricular hemorrhage	85	2.9	7	2.4	78	3.0	0.596
Microhemorrhage (diffuse axonal injury)	11	0.4	0	0.0	11	0.4	0.270
Patients with any intracranial hemorrhagic lesion	527	18.2	51	17.8	476	18.3	0.830
Acute traumatic lesion on head CT	633	21.9	53	18.5	580	22.2	0.138
Acute hemorrhagic lesion on head CT (no CSDH)	503	17.4	47	16.4	456	17.5	0.628

*CT, computed tomography; CSDH, chronic subdural hematoma*.

The use of serotonergic antidepressants is presented in Table 3. Selective serotonin reuptake inhibitors were the most commonly used antidepressants (7.2%). Among antidepressant using patients the most common agents were citalopram (30.0%) and escitalopram (25.8%).

**Table 3:** Antidepressant medication use in the cohort.

**Medication**	***n***	**%**
Any serotonergic medication	287	9.9
SSRI medication	209	7.2
Fluoxetine	15	0.5
Paroxetine	15	0.5
Sertraline	20	0.7
Citalopram	86	3.0
Escitalopram	74	2.6
SNRI medication	56	1.9
Duloxetine	27	0.9
Venlafaxine	29	1.0
TCA medication	31	1.1
Amitriptyline	30	1.0
Nortriptyline	0	0.0
Doxepine	0	0.0
Trimipramine	1	0.0

*SSRI, selective serotonin reuptake inhibitor; SNRI, serotonin–norepinephrine reuptake inhibitors; TCA, tricyclic antidepressant*.

The logistic regression analysis was calculated for SSRI medication, SNRI medication, TCA medication, and any serotonergic antidepressant. Only acute traumatic hemorrhagic lesions were included and chronic subdural hemorrhages were excluded. The results are shown in Table 4.

**Table 4:** Univariate and multivariate logistic regression analyses on CT-positive acute traumatic intracranial hemorrhage (no CSDH).

	**Univariate analysis**		**Multivariate analysis (*****n*** **=1,883)**							
			**SSRI**		**SNRI**		**TCA**		**Any serotonergic medication**	
**Variables in the model**	**OR (95% CI)**	***p***	**OR (95% CI)**	***p***	**OR (95% CI)**	***p***	**OR (95% CI)**	***p***	**OR (95% CI)**	***p***
SSRI medication	0.8 (0.5–1.2)	0.228	1.0 (0.6–1.7)	0.920	–	–	–	–	–	–
SNRI medication	1.8 (1.0–3.2)	0.065	–	–	1.1 (0.4–3.0)	0.910	–	–	–	–
TCA medication	0.7 (0.2–2.0)	0.508	–	–	–	–	0.3 (0.0–2.5)	0.256	–	–
Any serotonergic medication	0.9 (0.7–1.3)	0.628	–	–	–	–	–	–	0.9 (0.5–1.4)	0.602
GCS 13–14	6.6 (4.5–9.7)	<0.001	5.9 (4.0–8.7)	<0.001	5.9 (4.0–8.8)	<0.001	5.9 (4.0–8.7)	<0.001	5.9 (4.0–8.7)	<0.001
GCS <13	10.0 (7.4–13.5)	<0.001	8.9 (6.5–12.0)	<0.001	8.9 (6.5–12.0)	<0.001	8.9 (6.5–12.1)	<0.001	8.8 (6.5–12.0)	<0.001
Any antiplatelet medication	1.1 (0.9–1.5)	0.338	0.7 (0.4–1.0)	0.060	0.7 (0.4–1.0)	0.058	0.7 (0.4–1.0)	0.058	0.7 (0.4–1.0)	0.677
Any anticoagulation	1.7 (1.3–2.2)	<0.001	1.1 (0.8–1.7)	0.509	1.1 (0.8–1.7)	0.510	1.1 (0.8–1.7)	0.540	1.1 (0.8–1.7)	0.508
Male gender	1.5 (1.3–1.9)	<0.001	1.8 (1.3–2.4)	<0.001	1.8 (1.3–2.4)	<0.001	1.8 (1.3–2.4)	<0.001	1.8 (1.3–2.4)	<0.001
Age (years)	1.02 (1.01–1.02)	<0.001	1.02 (1.01–1.03)	<0.001	1.02 (1.01–1.03)	<0.001	1.02 (1.01–1.03)	<0.001	1.02 (1.01–1.03)	<0.001

*SSRI, selective serotonin reuptake inhibitor; SNRI, serotonin–norepinephrine reuptake inhibitors; TCA, tricyclic antidepressant; CT, computed tomography; CSDH, chronic subdural hematoma; GCS, Glasgow coma scale*.

Interactions between antithrombotic use and antidepressant use were analyzed with the two-way ANOVA. There was a statistically significant interaction with antiplatelet medication use and serotonergic antidepressant use (*p* = 0.006) but not with anticoagulant use and serotonergic antidepressant use (*p* = 0.590). Antiplatelet medication use was more common among patients taking serotonergic antidepressant medication (28.6 vs. 12.0%, *p* < 0.001).

In the univariate analysis, male gender, GCS under 15, older age, and anticoagulation were associated with an increased risk for an acute traumatic intracranial bleed. In the multivariate analyses, anticoagulation was not significantly associated with increased risk for bleeding. The number of patients was 1,883 in the multivariate analyses due to missing GCS data. In the multivariate analysis with the SSRI medication, the ORs for traumatic intracranial hemorrhage were non-significant with SSRI, SNRI, and TCA medication, and also with any serotonergic antidepressant.

## Discussion

There is minimal literature on the association between serotonergic antidepressant use and intracranial bleeding risk after head trauma. The rate of traumatic hemorrhagic lesions was similar in patients taking antidepressant medication vs. those not taking antidepressants. The risk for traumatic intracranial hemorrhage did not increase in those taking antidepressants, compared to those who were not, even when there was concomitant use of antithrombotic medication. These results are important given the high incidence of head injuries, the widespread use of serotonergic antidepressants medications, and the prior reports of serotonergic antidepressant-related systemic bleeding complications.

To our knowledge, the only study that has partly assessed the effects of antidepressant and traumatic intracranial bleeding has been conducted by Ibañez Pérez De La Blanca et al. (27). In that study, 504 older patients (≥60 years) with mild TBI were examined. Risk factors for traumatic intracranial lesions were analyzed with a multivariate logistic regression model. In that model, SSRIs were combined with benzodiazepines. Ibañez Pérez De La Blanca et al. concluded that SSRIs and/or benzodiazepines were protective for CT-positive intracranial lesions (OR = 1.681, 95% CI = 1.042–2.714, *p* = 0.033). The authors suggested that these drugs could possibly serve as neuroprotectors in elderly patients.

The use of antidepressants has increased over the years and is especially prevalent among the elderly (6). Moreover, TBI in the elderly is a growing public health concern (28). Advancing age (24) and antithrombotic agents (29, 30) are generally acknowledged risk factors for intracranial hemorrhage, both spontaneous and traumatic. From a pharmacological perspective, serotonergic medication could increase the likelihood of traumatic intracranial bleeding (9). The baseline risk of TBI-related intracranial bleeding that is associated with medications should be greatest among elderly patients on antithrombotic medication. Intuitively, the possible incremental bleeding risk associated with serotonergic antidepressants would manifest in elderly patients who use blood thinners. Based on the findings from the literature on spontaneous intracranial hemorrhage, the bleeding risk should be greatest among the patients using the antidepressants with the highest degree of serotonin reuptake inhibition (e.g., paroxetine, duloxetine, sertraline, escitalopram, fluoxetine) (31).

Inconsistent with our primary study hypothesis, serotonergic antidepressants were not associated with increased risk for traumatic intracranial bleeding. This null finding was consistent throughout our study as we analyzed different subgroups. In line with the literature, age was a risk factor for CT-positive traumatic intracranial hemorrhage in the combined sample. However, contrary to the general assumption, antiplatelet medication use did not increase the risk for traumatic intracranial bleeding in our study. It is worth noting that ASA is prescription-free in Finland, and some cases of usage of this medication are not recorded in the medical records. Thus, some ASA medication usage might not been detected by the researchers. It is well-known that the adherence to long-term medication is often poor, as the adherence rates average around 50% (32) and Finland is not an exception in this matter (33).

An interaction was found between antiplatelet medication and antidepressant use. Almost one third (28.6%) of the patients taking serotonergic antidepressant were also on antiplatelet medication. Even with this interaction, the incidence of intracranial bleeds was lower in antidepressant group, suggesting that this interaction did not affect the risk for intracranial bleeding.

Older patients have a high prevalence of heart and cardiovascular disease, and thus antithrombotic agents are more frequently used in this subpopulation to reduce the risk or to prevent the onset of thromboembolic events. Older age increases independently the risk of major hemorrhage, particularly intracranial hemorrhage, in patients with atrial fibrillation, whether or not they are taking warfarin (34). Age is strongly correlated with brain atrophy which may independently increase the risk for traumatic ICH, whether or not the patient is on an antithrombotic (35). It is possible that the effect of age itself confounds the effects of antithrombotic agents in our study.

Our current findings have clinical implications in relation to the acute management of patients with head injuries. In the emergency assessment of these patients, the risk of intracranial hemorrhagic complications is a paramount consideration. Age, preexisting diseases, medication, and injury characteristics influence the risk of hemorrhage. Decision-making on initial emergency head CT scanning, need for in-hospital monitoring, stratification for monitoring strategies, administration of prothrombotic agents to correct coagulation, and necessity of repeated head CT imaging is largely based on the presumed overall risk of traumatic intracranial hemorrhagic complications.

Deciding who should undergo head CT scanning after a head trauma is one of the most debated questions in emergency medicine, especially for those with clinical signs of mild TBI. In clinical practice, patients sustaining a mild TBI while on anticoagulation or on antiplatelet drugs are frequently automatically deemed to be at high risk of intracranial bleeding. The majority of international guidelines on the management of acute head injury do not advise specifically on the care of patients who are anticoagulated mainly due to the lack of sufficiently powered studies to address management in such subpopulations (1, 36). The National Institute of Clinical Excellence (NICE), National Emergency X-Radiology Utilization Study (NEXUS II), CT in Head Injury Patients (CHIP), American College of Emergency Physicians (ACEP), and the European Federation of Neurological Societies (EFNS) recommendations advocate that all patients taking warfarin should have an immediate CT scan irrespective of injury severity, GCS, or neurological symptoms (1, 3, 37–39). In this study, the risk of intracranial bleeding for those on anticoagulation was small and non-significant after adjustment for other factors. Additionally, serotonergic antidepressants did not increase the risk of traumatic intracranial hemorrhage irrespective of antiplatelets and anticoagulants. Our findings emphasize the importance and usefulness of other variables, such as the GCS and age, in the assessment and imaging triage of patients with head injuries.

This study has several strengths. The study sample is large, and the medication and CT imaging data is comprehensive. The study includes the whole severity spectrum of TBI from head injuries with no signs of TBI to severe TBI. Also, adult patients from all age groups in varying health conditions were included, although importantly our samples include a large percentage of older adults. The medication use of each patient was reviewed thoroughly. All CT findings, including hemorrhagic and other traumatic lesions were systematically coded. The cohort represents an unselected sample of patients with head injury who were consecutively treated in one ED. Other than requiring a clinically indicated head CT scan of the head, no eligibility criteria were applied, making the study findings more generalizable. All of the patients were treated in the same ED and the CT scans were interpreted by neuroradiologists.

There are also limitations in this study. First, the data were collected retrospectively from hospital records and hence some relevant information was missing. This may have biased our estimation of the relationship between patient and injury characteristics, and head CT findings. Second, we were unable to collect data on the exact dosage of the antidepressant medication. As noted above, there is some uncertainty on the use of ASA because this drug is prescription-free in Finland. Third, there were few users of direct oral anticoagulants because their use was uncommon during the period when most of the data were collected. Fourth, mechanism of injury is important in emergency medicine as part of clinical decision making regarding whether to order a head CT. We did not include mechanism of injury in our regression model because we could not, with reasonable confidence, differentiate high energy mechanisms (vs. medium or low), and some mechanisms had very small sample sizes and a sizeable minority were unknown. Finally, we do not have sufficient data (e.g., findings of repeat head CT imaging) on the radiological progression of the hemorrhagic lesions.

Future high-level observational studies (including antidepressant dosage) such as prospective cohort studies might allow better estimation of the potential risk of SSRI use and traumatic intracranial hemorrhage. The results of this study suggest that the use of serotonergic antidepressants by patients with head injuries does not warrant special precautions in the initial ED assessment.

## Conclusions

The use of serotonergic antidepressants was not associated with increased risk of intracranial hemorrhage after acute head trauma.

## Data Availability Statement

The statistical analyses and underlying data supporting the conclusions of this article will be made available by the authors to qualified researchers for research purposes, without undue reservation.

## Ethics Statement

The studies involving human participants were reviewed and approved by Ethics Committee of Pirkanmaa Hospital District, Tampere, Finland (identifier: R10027). Written informed consent from the participants' legal guardian/next of kin was not required to participate in this study in accordance with the national legislation and the institutional requirements.

## Author Contributions

HI wrote the statistical analysis plan, cleaned and analyzed the data, and drafted and revised the paper. GI, JP, JR, and JÖ contributed to drafting and revising the paper. AB and AK collected the imaging data and contributed to drafting and revising the paper. MN collected the medication data and contributed to drafting and revising the paper. TL wrote the statistical analysis plan, monitored data collection for the study, and contributed to drafting and revising the paper. All of the authors have approved the final version.

## Funding

TL and JP have received funding from Government's Special Financial Transfer tied to academic research in Health Sciences (Finland). JP was funded by the Academy of Finland (Grant #17379) and Maire Taponen Foundation. TL has received research grants from the Finnish Brain Foundation sr, the Emil Aaltonen Foundation sr, the Maire Taponen Foundation, the Science Fund of the City of Tampere, and the Finnish Medical Society Duodecim.

## Conflict of Interest

GI acknowledges unrestricted philanthropic support from the Mooney-Reed Charitable Foundation, Heinz Family Foundation, ImPACT Applications, Inc., and the Spaulding Research Institute. He serves as a strategic scientific advisor for NanoDX (formerly BioDirection, Inc.). JP has received speaker's fees from Orion corporation and Finnish Medical Association, and a travel grant from Stryker Corporation. JR has received speaker's fees from Orion Corporation, Bayer and Merck, and a travel grant from Boehringer Ingelheim. TL has received speaker's fees from Orion Corporation, Novartis Finland, and the Finnish Medical Society Duodecim. JR was employed by company Medbase Ltd. The remaining authors declare that the research was conducted in the absence of any commercial or financial relationships that could be construed as a potential conflict of interest.

## Publisher's Note

All claims expressed in this article are solely those of the authors and do not necessarily represent those of their affiliated organizations, or those of the publisher, the editors and the reviewers. Any product that may be evaluated in this article, or claim that may be made by its manufacturer, is not guaranteed or endorsed by the publisher.
